# The Future State of Newborn Stem Cell Banking

**DOI:** 10.3390/jcm8010117

**Published:** 2019-01-18

**Authors:** Katherine S. Brown, Mahendra S. Rao, Heather L. Brown

**Affiliations:** 1 Scientific and Medical Affairs, Cbr Systems Inc., 611 Gateway Blvd, Suite 820, South San Francisco, CA 94080, USA; hbrown@cordblood.com; 2 Collaborative Science Chair, InSTEM, Bangalore 560065, India; rao1789@gmail.com

**Keywords:** stem cell banking, newborn stem cells, perinatal stem cells, umbilical cord tissue, umbilical cord blood, placenta

## Abstract

Newborn stem cell banking began with the establishment of cord blood banks more than 25 years ago. Over the course of nearly three decades, there has been considerable evolution in the clinical application of stem cells isolated from newborn tissues. The industry now finds itself at an inflection point as personalized medicine and regenerative medicine continue to advance. In this review, we summarize our perspective on newborn stem cell banking in the context of the future potential that stem cells from perinatal tissues are likely to play in nascent applications. Specifically, we describe the relevance of newborn stem cell banking and how the cells stored can be utilized as starting material for the next generation of advanced cellular therapies and personalized medicine.

## 1. Current State of Newborn Stem Cell Banking

In September of 2018, the umbilical cord blood transplant and newborn stem cell banking communities celebrated the 30th anniversary of the first hematopoietic stem cell (HSC) transplant using cord blood as a graft for a patient with Fanconi’s anemia. The successful demonstration that cord blood is capable of reconstituting a patient’s blood and immune system, coupled with the confirmation that cord blood can be cryopreserved for later use, led to the establishment of cord blood banks, and thus the newborn stem cell banking industry, in the early 1990s [1]. Newborn stem cell banking encompasses public cord blood banks, which store cord blood units for use in an unrelated recipient; private banks, which store cord blood for future use by the donor or a first- or second-degree relative; and hybrid banks, which offer combined services [2]. It is estimated that more than more than 800,000 cord blood units are cryopreserved in public banks and over 5 million more are stored in private cord blood banks [3].

**Figure 1:**
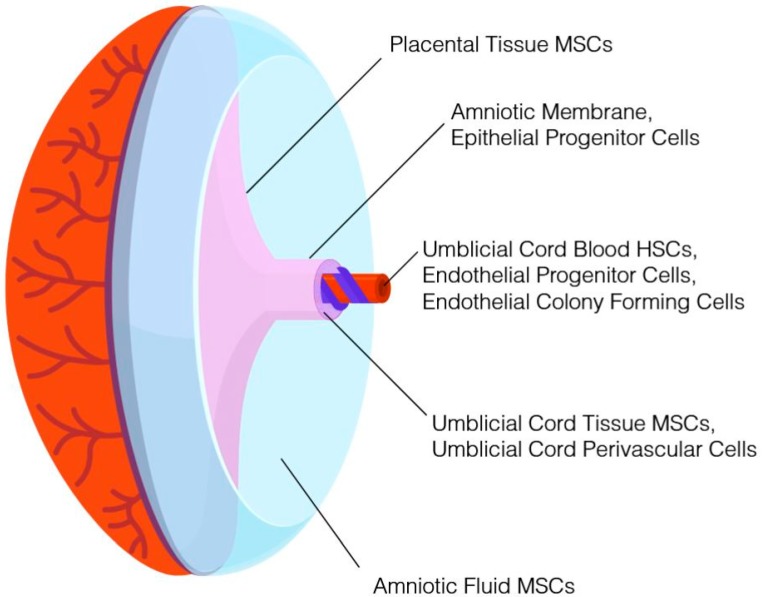
Schematic of newborn tissue that can be collected and stored for immediate or future use and the cell populations associated with each. Hematopoietic stem cells (HSCs) can be obtained from the umbilical cord blood, as can endothelial progenitor cells and endothelial colony-forming cells. Mesenchymal stem cells can be isolated from various locations within the placenta, umbilical cord tissue, amniotic membrane, and amniotic fluid. MSCs (mesenchymal stromal cells) can be obtained from umbilical cord blood, but successful isolation is time- and volume-dependent, rendering cord blood a less reliable source. The umbilical cord tissue is also a source of other stem or progenitor cells with potential applications.

It is widely recognized that additional perinatal tissues routinely discarded as medical waste contain nonhematopoietic cells with potential therapeutic value. For example, mesenchymal stromal cells (MSCs) can be isolated from placental tissue, umbilical cord tissue, and amniotic fluid. With the exception of amniotic fluid, which is obtained during an elective amniocentesis, these tissues are collected in a noninvasive procedure following birth of the neonate and would otherwise be discarded. Concomitant cryopreservation of multiple newborn tissues from the same donor (see, for example, [4]) has been demonstrated. Based on the potential therapeutic value, enhanced proliferative capacity, lack of ethical controversies, and reduced risk of exposure to virus and environmental toxins of newborn stem cells compared to stem cells from adult tissues, numerous cord blood banks expanded their processes in order to cryopreserve additional tissues alongside umbilical cord blood, and in some instances, as a stand-alone product. These new cryopreservation products encompass umbilical cord tissue, placental tissue, amniotic fluid, and amniotic membrane. In addition to serving as a repository of MSCs, epithelial cells and progenitor cells, endothelial cells and progenitor cells, and subpopulations of cells that may have therapeutic value can be isolated from the aforementioned tissues. A summary of perinatal tissues that can be cryopreserved and representative cell populations obtained from each are provided in Figure 1. Those families storing cord blood at a private bank in the United States typically pay between $300 and $2300 for the collection, processing, and initial storage, with annual storage fees thereafter [1]. Storage of an additional newborn tissue, such as umbilical cord tissue or placental tissue, costs an additional $800–1300. There is no charge to families donating newborn tissues, as public banks cover costs associated with collection, processing, and storage. The Parent’s Guide to Cord Blood Foundation [5] provides a global index of public and private banks and their respective services.

Certain maternal and neonatal parameters associated with cord blood quality, such as gestational age and birth weight, can be used by public banks to optimize donor selection in an effort to increase likelihood of utilization and as part of managing costs associated with tissue procurement [6]. The cellular content of cord blood is also influenced by seasonal variation and circadian oscillations; consideration of time-related parameters of cord blood collection is proposed as a mechanism to target cord blood donations with greater hematopoietic potential [7]. While the hematopoietic potential of cord blood units can be estimated by determining the number of cells expressing the CD34 antigen (CD34+ cells), different strategies must be employed by newborn stem cell banks to determine the potency of MSCs from perinatal tissues. Expression of cell surface markers is routinely used to identify an MSC population, yet is a poor substitute for product characterization [8]. For example, the maternal metabolic environment has been reported to alter bioenergetic profile as well as expression of proteins involved in stress response, metabolic, and cytoskeletal pathways of MSCs, confirmed by immunophenotype, from perinatal tissues [9,10]. Donor-to-donor variability of perinatal MSCs in anti-inflammatory and immunomodulatory assays has also been reported [11,12]. These findings are consistent with observations of donor heterogeneity of bone marrow MSCs and underscore the importance of functional assessments for newborn stem cell banks. Public banks exploring storage of allogeneic MSCs from newborn tissue may incorporate screening of donor cell lines for desired characteristics, such as immunomodulatory or angiogenic properties, prior to cryopreservation. A practical approach for private banks is to evaluate the post-thaw MSC product utilizing an assay that provides an estimate of functionality within the context of the intended therapeutic application. Incorporation of functional assessments by private banks is a logical extension of comparability studies described in more detail below. 

## 2. Newborn Stem Cells in Transplant and Regenerative Medicine Applications 

More than 40,000 hematopoietic stem cell transplants using cord blood have been performed during the last three decades [13]. In this setting, the hematopoietic stem cells in the cord blood are utilized for homologous reconstitution of the blood and immune system in the same manner as a bone marrow transplant. Cord blood is recognized as an alternative graft source for hematopoietic stem cell transplant in pediatric and adult patients and has been used in the treatment of over 80 diseases, including hematologic malignancies and disorders, congenital immunodeficiency disorders, and certain metabolic disorders [14]. 

There is also considerable interest in exploring cord blood as a therapeutic intervention in nonhematopoietic indications. In the mid-2000s, researchers began investigating cord blood in acquired neurological indications. Pilot and clinical trials enrolling pediatric patients with conditions such as cerebral palsy, autism spectrum disorder, and acquired hearing loss have confirmed the safety, and in some patients evidence of efficacy, of administering minimally manipulated cord blood cryopreserved in an autologous setting [15,16,17,18,19]. A small phase I study also confirmed the safety and feasibility of administering allogeneic unrelated cord blood to adult ischemic stroke patients [20]. Based on the observed safety profile and preliminary evidence of efficacy, additional studies to determine efficacy and to evaluate the safety of the approach in human leukocyte antigen (HLA)-matched related and unrelated donor cord blood are either underway or planned [16,17,20]. 

**Figure 2:**
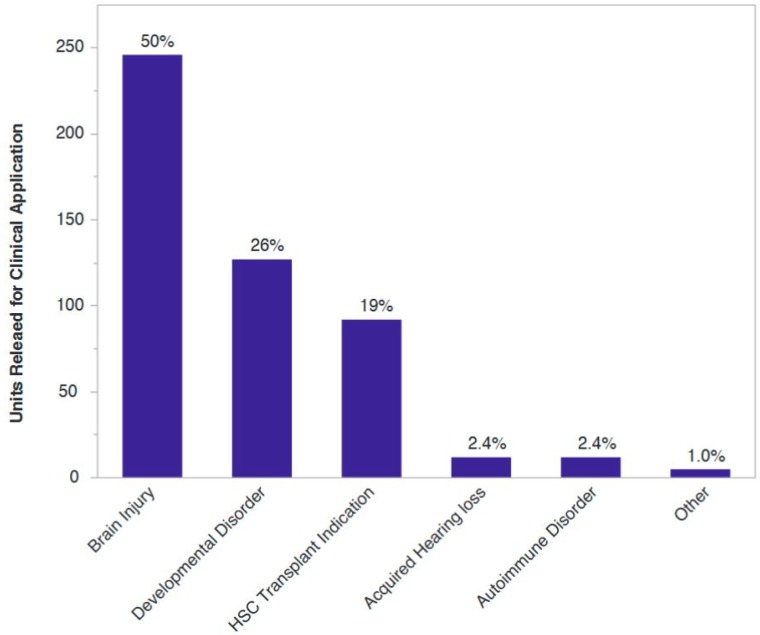
Categories of clinical applications for which cord blood units have been released from a private newborn stem cell bank. As of December 2018, the Cord Blood Registry has released over 500 cord blood units for use in clinical applications, slightly less than 20% of which were utilized in a hematopoietic stem cell transplant. Data are presented as the percentage of units released for each generalized category.

A recent review of clinical trials employing perinatal tissue-derived products in advanced cell therapy identified 281 clinical studies registered between 2005 and 2015, and acquired neurological conditions or disorders was the second most common category of diagnosis behind trials in hematology or oncology with manipulated cell types [21]. Of more than 500 cord blood units released for clinical application from our institution, 80% have gone to clinical trials or experimental uses in regenerative medicine, with the vast majority of those indications being neurological injuries sustained at or around the time of birth or diagnosis associated with said injuries (Figure 2). 

Cryopreserved perinatal tissues are also being explored for their capacity to augment established uses of cord blood in traditional transplant medicine. For example, monocytes isolated from cryopreserved cord blood are used to manufacture a cell therapy product aimed to augment cord blood transplantation in the setting of inherited demyelinating conditions of the central nervous system [22]. Furthermore, the potential for MSCs isolated from cryopreserved cord tissue or placental tissue to facilitate ex vivo expansion of cord blood hematopoietic stem cells has been reported and provides further rationale for storing multiple newborn tissues from the same donor. 

Interest in exploring cryopreserved newborn stem cells in regenerative applications has continued to increase over the past decade. The mechanism by which the therapeutic cells exert their effects in many of these exploratory studies is theorized to involve immunomodulation and paracrine-based signaling facilitating endogenous tissue repair rather than direct cell replacement or engraftment. The evolving landscape of clinical trials in regenerative medicine utilizing umbilical cord tissue, placental tissue, and minimally manipulated cord blood has been recently reviewed in depth by others, as have approaches to ex vivo cord blood expansion and efforts to improve outcomes following cord blood stem cell transplantation in adult recipients (see, for example, [2,13,23,24]). The remainder of this discussion will focus on the application of cryopreserved perinatal cells in more nascent technologies.

## 3. Emerging Advanced Cellular Therapies and Changes to the Business Model

Cord blood has established utility in the setting of hematopoietic stem cell transplant medicine, and cord blood stem cells are also being investigated for their ability to induce healing and repair tissue, which has the potential to greatly increase the utilization of cord blood in the clinical setting. Nevertheless, both private and public institutions within the newborn stem cell banking industry face mounting challenges.

Public cord blood banks have faced increased regulations over the past decade. In particular, in 2007, the FDA established that cord blood, unlike bone marrow, intended for use in an unrelated recipient is a processed, prescription product, or drug. Effective as of 2009, public banks are considered a “manufacturer” and are required to have approval from the FDA biologics license application (BLA) for cord blood. The public banking community has repeatedly pointed out that the process for achieving licensure is both onerous and costly, negatively impacting the costs of collecting, storing, and distributing cord blood units [25]. One factor influencing treating physicians is that many adult patients require two cord blood units to meet the cell dose thresholds for a hematopoietic stem cell transplant, adding complexity to the transplant itself and pushing cord blood towards being cost-prohibitive compared to other graft sources. To date, only seven public banks have successfully obtained licensure, while the remaining public banks are allowed to continue operations as they move towards compliance. Public banks recover costs when cord blood units are released from inventory, not at the time of cryopreservation. Additionally, public banks have a relatively low utilization rate, a significant contributor considering that nearly 90% of institutions have reported struggling financially [26]. Lastly, successful licensure is not retroactive for previously collected inventory; units collected pre-licensure, while theoretically of equivalent quality to licensed units, can only be used under an investigational new drug (IND) application, which are granted for specific uses.

With a positive safety profile to date, the use of cord blood cells in regenerative medicine applications appears poised to increase the number of clinical settings in which the cells can be considered as part of a therapeutic intervention, as discussed above. This rapid evolution for potential indications outside of hematopoietic reconstitution has the potential to greatly influence the utilization rate of cord blood units from the public inventory. Each bank, though, would need to be approved for releasing cord blood units for new indications by performing the requisite clinical studies, which public banks have neither the resources nor the commercial initiative to do. Private cord blood banks are indirectly affected by this issue due to a lack of comparability studies. To our knowledge, only one controlled study in a regenerative medicine application has directly compared infusion of autologous cord blood to allogeneic unrelated cord blood from a public cord blood bank [27]. Should allogeneic unrelated cord blood prove to be therapeutically relevant in a regenerative medicine setting, for example, patients with autism spectrum disorder (ASD), a public bank could consider applying for a BLA for use in ASD, which would be separate from the BLA for uses in transplant medicine. This process would establish for the public banks another avenue for cord blood unit utilization and revenue upon unit release. For private cord blood banks, where revenue is recognized at the time of storage rather than release, there is less motivation to invest in clinical trials as cord blood units could be released under an IND held by the treating facility. The dilemma then is that private institutions, which are best suited to commit financially to exploring new indications, have the least motivation to do so from the perspective of short-term revenue generation. 

Commercial institutions in adjacent business areas are likely to influence the near-term financial sustainability of public cord blood banks. Companies such as Gamida Cell Ltd, Fate Therapeutics, and others are focused on expansion technologies and approaches for improving the efficiency of homing and engraftment of cord blood stem cells. While applicable to both private and publicly banked units, public banks are likely to benefit most from successful clinical translation if these technologies increase utilization of units that would otherwise fail to meet cell dose thresholds. Nohla Therapeutics is taking a different approach by developing off-the-shelf, ex vivo expanded products from cord blood units to provide a short-term hematopoietic bridge following transplant of an unmanipulated cord blood graft. If successful, Nohla’s product could help improve outcomes in patients undergoing a cord blood transplant. These technologies address current challenges in cord blood hematopoietic stem cell transplantation and their success could increase utilization rate from the public inventory, which in turn would help bolster the financial health of public banks. 

Public cord blood banks also have the opportunity to diversify revenue streams through creative outsourcing of inventory that is unlikely to be used as a graft for hematopoietic reconstitution. Blood banks collecting and distributing peripheral blood for use in transfusion medicine have already begun a similar shift as they look to capitalize on ancillary material or products that have expired. Likewise, the increasing demand for human platelet lysate as a supplement for use in routine cell culture has led to a niche market for blood banks. Public cord blood banks are exploring the applicability of donated cord blood in nascent technologies, such as chimeric antigen receptor T-cell (CAR-T) products. Importantly, a single cord blood unit is often sufficient for CAR-T, and for these and other similar immunotherapies, the cells are only required for a short time. Because units are HLA-typed at the time of processing, there is also a great deal of interest in utilizing inventory from public cord blood banks to create a haplobank of HLA-homozygous induced pluripotent stem cells (iPSCs); proof-of-concept studies have demonstrated the feasibility of this approach for populations in various geographical locations [28]. 

Private newborn stem cell banks are also interested in exploring the application of cord blood in the evolving field of immunotherapies and more than minimally manipulated products. Clinical studies initiated in the mid-2000s sought to determine the potential of nonmanipulated cryopreserved cord blood in Type 1 diabetes. Consistent with the results of clinical trials in acquired neurological injuries, autologous infusion of cord blood in patients with Type 1 diabetes was confirmed to be safe and feasible both alone and when administered alongside daily vitamin D and docosahexaenoic acid [29,30]. Investigators postulated that the limited number of regulatory T cells in cord blood limited the potential for sustained preservation of C-peptide, raising the possibility that infusion of regulatory T cells isolated and expanded from cord blood may be more efficacious than the heterogeneous cell populations in nonmanipulated cord blood [30]. The feasibility of expanding regulatory T cells from privately banked cord blood in a current good manufacturing practice (cGMP) setting was recently confirmed, opening the door for the approach to be explored in a clinical trial setting for Type 1 diabetes and other autoimmune conditions [31]. It is also worth noting that with the refinement in the safety and efficiency of gene editing capabilities and early successes in gene therapy clinical trials, one can foresee cord blood collection and banking in a private setting with the future intent of personalized, autologous gene therapy for individuals with a known genetic disorder in the future. Private banks have also shown interest in leveraging the technical advancements in iPSC reprogramming on behalf of their clients. In a private bank setting, starting material can be used to generate personalized, donor-specific iPSCs for autologous use. In fact, cells from cord blood and cord tissue from the same donor stored at a private bank can be utilized to generate iPSCs; confirmation that lines generated from either source material are of equivalent quality provided rationale for utilizing the cord tissue cells as starting material, preserving the cord blood unit in its entirety for future clinical utility [32]. Additionally, MSCs isolated from previously cryopreserved cord tissue at a private bank are amenable to reprogramming with multiple integration free methods on semi- or fully automated technology platforms for enhanced standardization and scalability [33,34]. One potential business model would be to utilize a portion of collected newborn material, either cord blood or cord tissue, to generate a biologically potent, individualized iPSC line which is then stored as a companion product for future potential uses. Extracellular vesicles, including exosomes, represent another intriguing potential companion storage offering. A number of commercial institutions have established off-the-shelf stem cell-derived extracellular vesicle products and are moving them into clinical trials. It will be interesting to see what influence, if any, the burgeoning field of cell-free therapeutics has on the newborn stem cell banking industry.

There is increased clarity on potential application of privately banked stem cells outside of the established uses in HSC transplants. Early recognition of the interest and anticipated utilization of MSCs across a variety of clinical settings garnered the interest of the private banks as an opportunity to leverage infrastructure and technology platforms to provide storage of umbilical cord tissue as a service under the collection and manufacturing model already established as part of private cord blood banking. Similarly, private institutions led the industry in the banking of placental tissue. Although efforts are underway (see, for example, [35]), public banks have been slower to establish the same programs, as the path for and extent of reimbursement will remain undefined until there are established and more widely practiced clinical applications for cells derived from these alternative perinatal tissues. 

For cord blood, clinical outcomes of transplants are influenced by the graft characteristics, including nucleated cell dose, stem cell dose, and HLA match, while the impact of the volume reduction processing technology used in preparing cord blood for cryopreservation is less evident, assuming that units selected meet appropriate criteria for the individual recipient and appropriate methods for thawing are followed [36,37]. While cryopreservation of umbilical cord tissue and placenta as source material have been an integral part of stimulating new avenues of clinical research, their rapid adoption led to a variety of different approaches for preparation and cryopreservation. Thus, private newborn stem cell banks and some public banks find themselves in a position where similar material is stored, but comparability of the final product has yet to be determined. The industry clearly finds itself at a point where comparability studies, to confirm that products processed and stored via any number of processes, are increasingly warranted. Cord tissue and placental tissue can be prepared for cryopreservation as either a cell suspension or composite material thawed at a later date for isolation of cells. Thus, assays for functional attributes of the final product after thawing of a cell suspension, or in the case of whole tissue cryopreservation, recovery of cells from thawed composite material, are a logical point for implementing a standardized approach to determine comparability. Importantly, assessments should ideally encompass functional attributes associated with mechanisms of action, rather than simply identity and purity of the cell population. These studies require investment from private institutions, but are well justified given the benefit to the client as well as scientific and medical communities.

## 4. Changing Landscape of the Newborn Stem Cell Banking Market

The proliferation of private cord blood banks in the early 2000s led to market saturation in many geographical regions. Mergers and acquisitions subsequently led to consolidation in the private banking industry. It is estimated that over the last decade, the number of European banks decreased by one-third through consolidation activity [38]. The merger of Cell Care Australia, the largest private newborn bank in Australia, with Insception Lifebank, the largest private bank in Canada, exemplify industry willingness to explore major intercontinental activity. Although a relatively late arrival to the newborn stem cell banking landscape, India is poised to overtake the market for cord blood banking and families have the opportunity to choose from any number of different banks. One of those options is Cryo-Save, which leverages processing capabilities and economies of scale by operating only several centralized processing facilities in Europe, India, and South Africa, with numerous regional facilities operating under the Cryo-Save trademark through licensing agreements. In stark contrast, the government of China allows only a single cord blood bank to operate in each province; although consumer choice is limited, each licensed bank must function as a hybrid bank, providing donation-based and private storage services. 

Hybrid banks were originally met with resistance from the banking community based on perceived conflicts of interest for the donor. More recently, there has been greater acceptance of the hybrid model, with several institutions demonstrating that private banking can be used to offset costs for altruistic public donations without deterring from the donor pool. Companies such as StemCyte International have found stability in the consumer market while also providing donated cord blood units for use in unrelated recipients. As evidenced by the acquisition of the hybrid bank CORD:USE by the private bank Cryo-Cell International, mergers and acquisitions also present an opportunity to enter into new market sectors, such as public banking, while limiting the potential financial risks of de novo development. Some industry activities meanwhile capitalize on infrastructure expertise and capacity for biobanking as a logical extension of services. For example, Cord Blood Registry was acquired and merged with California Cryobank. The newly established California Cryobank Life Sciences Platform provides newborn stem cell banking alongside reproductive tissue services. Celularity Inc., a spinout of Celgene, is developing placenta-derived allogeneic immuno-oncology and regenerative products while offering cryopreservation of umbilical cord and placental tissues through the private arm LifeBank USA. Celularity also acquired CariCord, a private cord blood bank affiliated with ClinImmune laboratories, presumably in part to augment biosourcing capabilities for future placenta-derived products. 

## 5. Conclusion

Within the United States, the health care system is critical to long-term economic stability for newborn stem cell banking. Health care coverage of the costs associated with collection and cryopreservation of newborn tissues for future use and establishing insurance reimbursement once clinical efficacy and cost calculations are established in regenerative medicine applications would provide for economic incentives for all invested parties and help the industry meet the increasing demand for precision health care. Outside of establishing legislation mandating education by health care providers for expecting families, efforts to lobby Congress have thus far been unsuccessful. The Cord Blood Association, a nonprofit organization comprised of stakeholders across the banking industry, advocates on behalf of the community to advance relevant legislation and modifications to the regulatory framework and may be a more effective approach than previous efforts. 

**Figure 3:**
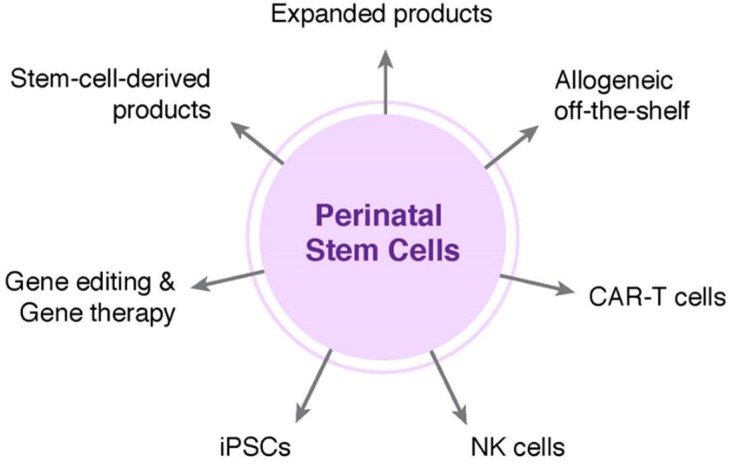
Newborn stem cells in emerging and advanced cellular therapies. Stem or progenitor cells obtained from various newborn tissues are depicted in the center, while potential downstream products are represented on the periphery. iPSCs, induced pluripotent stem cells; NK cells, natural killer cells; CAR-T cells, chimeric antigen receptor T cells.

Emerging technologies have the potential to influence the direction of newborn stem cell banking; both public and private banking institutions will need to identify a strategic path in order to position themselves favorably for the long term. Public and private banks are actively exploring ways to augment their model from providing storage of a minimally manipulated cellular product to one that recognizes the promise in companion products and provides starting material for downstream applications (Figure 3). It is also clear that private and public cord blood banks will need to react differently to the financial and industry challenges, based on the divergence in their models and how regulations are applied to each.
